# The Role of Notch3 Signaling in Kidney Disease

**DOI:** 10.1155/2020/1809408

**Published:** 2020-10-22

**Authors:** Cheng Yuan, Lihua Ni, Changjiang Zhang, Xiaoyan Wu

**Affiliations:** ^1^Department of Gynecological Oncology, Zhongnan Hospital of Wuhan University, Wuhan 430071, China; ^2^Department of Nephrology, Zhongnan Hospital of Wuhan University, Wuhan 430071, China; ^3^Department of Cardiology, Renmin Hospital of Wuhan University, Wuhan 430060, China; ^4^Cardiovascular Research Institute, Wuhan University, Wuhan 430060, China; ^5^Hubei Key Laboratory of Cardiology, Wuhan 430060, China

## Abstract

Notch receptors are transmembrane proteins that are members of the epidermal growth factor-like family. These receptors are widely expressed on the cell surface and are highly conserved. Binding to ligands on adjacent cells results in cleavage of these receptors, and their intracellular domains translocate into the nucleus, where target gene transcription is initiated. In the mammalian kidney, Notch receptors are activated during nephrogenesis and become silenced in the normal kidney after birth. Reactivation of Notch signaling in the adult kidney could be due to the genetic activation of Notch signaling or kidney injury. Notch3 is a mammalian heterodimeric transmembrane receptor in the *Notch* gene family. Notch3 activation is significantly increased in various glomerular diseases, renal tubulointerstitial diseases, glomerular sclerosis, and renal fibrosis and mediates disease occurrence and development. Here, we discuss numerous recently published papers describing the role of Notch3 signaling in kidney disease.

## 1. Introduction

Notch receptors are highly conserved signaling molecules that are widely distributed on the surface of a variety of cells. Notch receptors are activated by interactions with ligands on adjacent cells and play important roles in cell fate decisions and differentiation in many tissues during embryonic and postnatal development [1]. Four receptors have been identified in mammals and are designated Notch1-Notch4. The abnormal activation of these receptors mediates the occurrence and development of various diseases, such as fatty liver disease [2], skeletal disease [3], renal fibrosis [4], and malignant tumors [5]. Currently, research on the mechanism by which Notch receptors mediate nonneoplastic nephropathy focuses mainly on the Notch1 and Notch2 subtypes. However, recent studies [6–9] have shown that Notch3 is abnormally activated, plays important roles in a variety of glomerular and tubulointerstitial diseases, and directly affects the prognosis and outcome of nephropathy. This review summarizes the recent advances in the understanding of Notch3 in nonneoplastic kidney diseases.

## 2. Notch Receptors and Their Ligands

**Figure 1:**
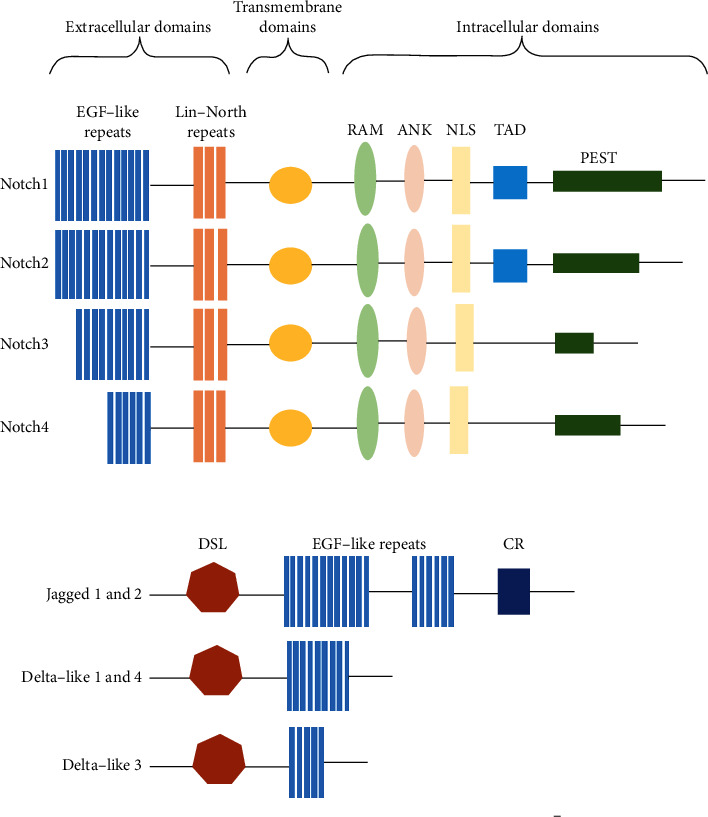
Schematic of Notch receptors and ligand families. The Notch signaling pathway includes four Notch receptors (Notch1, 2, 3 and 4) and five canonical ligands (Jagged 1 and 2, Delta-like 1 and 4, and Delta-like 3). Notch receptors comprise extracellular domains, transmembrane domains, and intracellular domains. The ligands of Notch receptors can be divided into two groups according to the length and subtype of the EGF-like repeats. RAM: Rbpj interacting domain; ANK: ankyrin repeat; NLS: nuclear localization signal; TAD: transcriptional activator domain; PEST domain: proline-, glutamine-, serine-, and threonine-rich domain; DSL: Delta-Serrate-Lag2; CR: cysteine-rich region.

The Notch signaling pathway is composed of Notch receptors, ligands, and downstream transcription factors. Binding of ligands to Notch receptors activates downstream target genes and exerts various biological effects (Figure 1).

Notch receptors are highly conserved type I transmembrane glycoproteins that contain extracellular domains, transmembrane segments, and intracellular segments [10–12]. The Notch extracellular domain (NECD) contains epidermal growth factor- (EGF-) like repeats and three Lin-Notch repeats. The NECD mediates the release of the intracellular segment, the Notch intracellular domain (NICD), which contains the Rbpj-interacting domain (RAM), ankyrin repeats (ANKs), the transcriptional activator domain (TAD), nuclear localization signals (NLSs), and a proline-, glutamate-, serine-, and threonine-rich domain (PEST domain). The mammalian Notch receptor is divided into four subtypes depending on the length of the extracellular EGF-like repeats and the conserved PEST motif at the end of the intracellular segment: Notch1-Notch4. Notch1 and Notch2 have 36 EGF-like repeats, Notch3 has 34, and Notch4 has 29; the PEST domain of Notch1 is the longest, the PEST domain of Notch3 is the shortest, and the PEST domain of Notch2 and Notch4 is an intermediate length. Differences in the molecular structure result in the different Notch receptor subtypes, which subsequently mediate different signaling pathways. In addition, Notch1 and Notch 2 contain a TAD, but Notch 3 and Notch4 do not. The TAD contains phosphorylation sites that allow other signaling pathways to modulate the Notch activity. The TAD of Notch3 is different from the TADs of Notch1 and Notch2 [13]. Compared with the extracellular domains of the other Notch receptors, the extracellular domain of Notch3 lacks EGF-like repeat 21 and some segments of sequences 2 and 3. Thus, Notch3 is expressed only in vascular smooth muscle cells (VSMCs), the central nervous system, and thymus cell subsets in adult individuals.

Notch receptors can be activated by interaction of the NECD with a canonical or noncanonical Notch ligand. The canonical Notch ligands belong to the Delta, Serrate, and LAG-2 (DSL) family and include Jagged1 (Jag1), Jagged 2 (Jag2), and delta-like protein (DLL) 1, DLL3, and DLL4. Noncanonical Notch ligands include Notch-like epidermal growth factor-related receptor (DNER), delta-like homolog protein (DLK)1, DLK2, and soluble Y-box protein-1 (YBX1).

## 3. Notch Signaling

**Figure 2:**
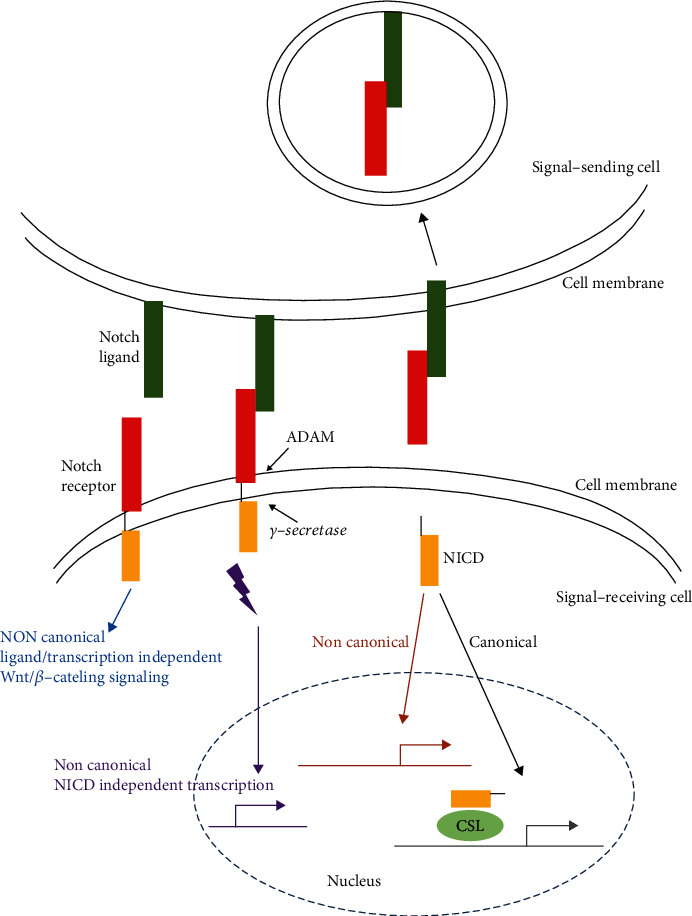
Schematic of Notch signaling. Notch receptor occupation by ligands promotes proteolytic changes via *γ*-secretase and ADAM. Proteolytic cleavage of the receptor releases the NICD. The NICD targets the nucleus and promotes transcription by binding to the CSL transcription factor in the recipient cell. Endocytosis of the ligand/NECD complex can contribute to the Notch activation via dissociation of the NECD from the Notch heterodimer. NECD: Notch extracellular domain; NICD: Notch intracellular domain; CSL: CBF-1, suppressor of hairless, Lag; ADAM: a disintegrin and metalloprotease.

The Notch signaling activation is mediated by interactions between Notch receptors and their ligands. This binding triggers Notch cleavage by complexes formed by a disintegrin and metalloprotease (ADAM) and *γ*-secretase, which ultimately leads to release of the NICD (Figure 2). As an active form of Notch, the NICD can be translocated to the nucleus via endocytosis and vesicular trafficking and ultimately activate canonical and noncanonical transcriptional complexes.

The canonical transcriptional activation complexes include the NICD and the transcription factor CSL, which is bound to DNA [14]. CSL comprises C promoter binding factor-1 (*CBF-1*) and suppressor of hairless and lymphocyte-activation gene-1 (*LAG-1*). Binding of the NICD to CSL leads to the transcriptional activation of potential target genes, while in the absence of the NICD, CSL acts as a transcriptional repressor.

Noncanonical Notch signaling can also activate the target gene transcription in the presence or absence of the NICD [15]. Additionally, Notch signaling can posttranslationally target Wnt/*β*-catenin signaling independent of ligand binding or transcription [16]. The biological effects mediated by the Notch signaling pathway are closely related to the cell type, expression of Notch receptor subtypes, and the cellular microenvironment.

## 4. Notch Signaling and the Kidney

The Notch signaling pathway is involved throughout the process of kidney development [17]. As the kidney matures, the expression of Notch signaling components gradually decreases and even disappears. The adult kidney exhibits extremely low expression of the receptors Notch1/2 and the ligand Delta [18]. Notch3 is expressed only in renal VSMCs [19], and its expression is not detectable in glomerular or tubular epithelial cells [20]. Recent studies have proven that the Notch1 activation contributes to the development of albuminuria, glomerulosclerosis, and kidney dysfunction and mediates renal fibrosis in vivo and in vitro [21, 22]. Notch1 inhibition alleviates diabetic kidney disease, nephrotic syndrome, and fibrosis [22, 23]. In addition, Notch2 deletion from epithelial renal epithelial precursors severely compromises renal development, with loss of the proximal epithelium [24]. Notch2 mutations have also been associated with an Alagille-like phenotype in humans, who present with renal abnormalities [25]. A previous study showed that the overexpression of Notch4 enhances the fibrotic activity of TGF-*β* in a human-derived kidney tubule cell line, and that the tubular Notch4 expression is augmented in fibrotic kidneys from diabetic mice and humans [26]. In addition, the Notch4 expression was found to be upregulated in various renal cells in patients with HIV-associated nephropathy (HIVAN) and in rodent models of HIVAN [27]. Glomerular sclerosis and severe proteinuria appear in 2-week-old NICD transgenic mice [28], while Notch3-knockout mice are protected from tubular injury and interstitial collagen deposition induced by unilateral ureteral obstruction (UUO) [29]. Thus, an enhanced understanding of Notch3 may be beneficial for the development of therapeutic options to delay the progression of kidney disease.

## 5. Notch3 and Renovascular Disease

Notch3 is expressed in VSMCs in various organs and plays an important role in maintaining the structural integrity and functional stability of arteries, especially cerebral and renal arteries. To date, the only available data linking Notch3 to vascular disease comes from patients with cerebral autosomal dominant arteriopathy with subcortical infarcts and leukoencephalopathy (CADASIL) [30]. Mutations in the extracellular domain of Notch3 in VSMCs mediate the pathology of CADASIL. Interestingly, patients with CADASIL often have renal arteriosclerosis, renal fibrosis, glomerular enlargement, and chronic kidney disease [31]. Furthermore, compared to wild-type mice, Notch3-/- mice were found to have thinner afferent renal arterioles and to exhibit compromised renovascular reactivity in a model of hypertension induced by acute administration of norepinephrine or angiotensin II [32]. However, protective effects were observed when wild-type mice were treated with a *γ*-secretase inhibitor (a Notch signaling suppressor) [32]. In other words, Notch3 deletion affects the renovascular response. The deficient response may be attributed to a structural defect that occurred during the development of the vascular wall, and the RhoA/Rho kinase pathway might be involved in these processes [33].

## 6. Notch3 and Renal Glomerular Disease

Among patients with end-stage kidney disease, 90% have glomerular disease. Podocyte injury (such as apoptosis, death and decreased numbers) and parietal epithelial cell (PEC) activation are common factors determining the progression of glomerular disease.

### 6.1. Podocytes

The Notch3 expression is undetectable in the glomeruli of normal kidneys but is evident in injured podocytes. Notch3 is highly expressed in the nucleus and cytoplasm of podocytes in kidney biopsies of patients with focal segmental glomerulosclerosis (FSGS) and lupus nephritis (LN) [34]. Notch3 was significantly activated in podocytes in mouse models of FSGS induced by adriamycin injection, and the model animals exhibited increased proteinuria, decreased podocyte numbers, and glomerulosclerosis [34]. Notch inhibition alleviated these changes. Similarly, the abnormal activation of Notch3 in podocytes induced phenotypic changes associated with cell migration, inflammation, proteinuria, and loss of renal function in an animal model of rapidly progressive renal disease induced by administration of nephrotoxic sheep serum [20]. In contrast, Notch3-knockout mice and wild-type mice treated with a specific Notch3 inhibitor were protected from the decline in renal function. These protective effects may be associated with activation of the NF-*κ*B pathway, especially the p65 subunit, in podocytes.

In vivo studies [20, 34] demonstrated that the Notch3 activation in podocytes (via infection with adenovirus expressing a constitutively active Notch3 intracellular domain) led to alterations in cell shape (elongation), acquisition of a migratory phenotype, downregulated transcription of p21 and p27 (which promote cell cycle progression), and upregulation of Aurora Kinase B. Unfortunately, aberrantly expressed Aurora Kinase B could induce cell cycle progression but could not support the assembly of a functional mitotic spindle, which induced cell death by mitotic catastrophe.

### 6.2. PECs

A descriptive analysis [35] of aged (27 months) and young (3 months) mice was conducted to study the effects of aging on glomerular PECs. The results demonstrated that the PEC density was markedly lower in aged mice than in young mice. Additionally, increased glomerulosclerosis, extracellular matrix production by PECs, numbers of activated and profibrotic PECs, expression levels of epithelial-mesenchymal transition (EMT) markers (*α*-SMA and vimentin), and activation of Notch3 were observed in PECs from aged mice. The response of PECs to glomerular injury can involve a common pathway that induces fibrogenesis after podocyte loss, which typifies glomerular disorders. Reducing mammalian target of rapamycin (mTOR) levels was found to increase the PEC density and PEC-derived crescent formation in a rat model of acute podocyte depletion and in aged kidneys [36]. These changes induced by mTOR inhibition might be associated with increased levels of EMT markers (platelet-derived growth factor (PDGF) receptor-*β* and *α*-SMA) and activation of Notch3 and PECs.

In conclusion, Notch3 inhibition might protect podocytes and decrease the PEC activation, suggesting a novel therapeutic strategy for FSGS and rapidly progressive glomerulonephritis (RPGN).

### 6.3. Mesangial Cells

Mesangial cells originate from the metanephric mesenchyme and maintain the structural integrity of the glomerular microvascular bed and homeostasis of the mesangial matrix [37]. In response to metabolic, immunologic, or hemodynamic injury, these cells undergo apoptosis, acquire an activated phenotype, and undergo hypertrophy and proliferation with excessive production of matrix proteins, growth factors, chemokines, and cytokines. Mesangial cells are primary targets of immune-mediated glomerular diseases such as IgA nephropathy, membranoproliferative glomerulonephritis, and metabolic diseases (such as diabetes). The abnormal activation of Notch3 is closely associated with these diseases.

An in vivo study [38] indicated that in a rodent model exhibiting similarities to human mesangioproliferative diseases, the expression levels of Notch3 and its downstream target genes (HES2 and Hey-2) were significantly increased compared with those in the control animals; interestingly, peak mesangial cell proliferation coincided with the maximal Notch3 expression. The immunohistochemical staining results showed that the healthy human tissue did not exhibit positive staining for Notch3 except in VSMCs; however, in IgA nephropathy, Notch3 staining was increased in glomerular cells, predominantly within the mesangial cells [29].

The expression of Notch3, Jagged2, and the cold shock protein YBX-1 was found to be upregulated in mesangial cells after exposure to TGF-*β*1. Similar upregulation of Notch3, Jagged2, and YBX1 was also observed in glomeruli isolated from patients with IgA nephropathy. Pearson correlation analyses proved that the glomerular expression of Notch3 was positively correlated with that of both Jagged and YBX1 [29]. Jagged2 is a canonical Notch ligand [39], and YBX-1, a newly identified noncanonical ligand within the Notch3 signaling framework, is characterized as a downstream target in PDGF-BB (a key cytokine that drives mesangioproliferative diseases) signaling [39–41]. In addition, temporally and spatially coordinated upregulation of the receptor Notch3 and the noncanonical ligand YBX-1 within the glomerular compartment was observed in a rat model of mesangioproliferative nephritis [42]. Raffetseder et al. [38] demonstrated that extracellular YBX-1 associates with and activates Notch3 in rat mesangial cells; inhibition of this activation by blockade/depletion of extracellular YBX-1 led to the increased expression of cellular Notch3 accompanied by activation of downstream target genes (HES2 and Hey-2) and YBX-1 upregulation. In inflammatory mesangioproliferative diseases, blockade of ligand binding may be hindered by this autoregulatory loop.

## 7. Notch3 and Tubulointerstitial Diseases

Tubulointerstitial nephritis is characterized histologically by inflammatory changes, extracellular matrix protein accumulation, tubular dilation, and atrophy [43].

### 7.1. Acute Kidney Injury (AKI)

AKI is often associated with the use of drugs, such as *β*-lactam antibiotics and nonsteroidal anti-inflammatory drugs, and is likely mediated by allergic mechanisms [44–46]. Kramer et al. [47] suggested that the expression of Notch1-3, Notch ligands (Jagged1, Jagged 2, and DLL4), and Notch target genes (HES1, Hey2, HeyL, Sox9, and PDGF receptor *β*) was increased in a mouse model of ischemia and reperfusion; however, treatment with a *γ*-secretase inhibitor (DBZ) blocked Notch signaling and specifically downregulated the expression of Notch3 (although no difference in the expression of Notch2 or Notch4 was observed) and Notch target genes (HES1, Hey2, HeyL, and PDGF receptor *β*) in ischemic mice. Additionally, the ischemic mice exhibited reduced interstitial edema and altered interstitial inflammation patterns after the DBZ administration. Yuan et al. demonstrated that Notch3 was upregulated in the kidneys of lipopolysaccharide- (LPS-) induced mice and in LPS-treated TCMK-1 renal epithelial cells [48]. In addition, they found that LPS-induced AKI in mice and the inhibition of cell growth, promotion of lactate dehydrogenase production, and increase in the proportion of apoptotic cells were accompanied by upregulation of the Notch3 expression in vivo and in vitro. Targeting Notch3 could thus alleviate LPS-induced renal cell dysfunction. These data suggest that blocking Notch3 ameliorates experimental murine AKI.

Kavvadas et al. [8] demonstrated that compared to control mice, mice with activated Notch3 signaling in renal epithelial cells exhibited exacerbated inflammatory cell infiltration and severe tubular damage after ischemia reperfusion. However, Notch3-knockout mice were protected against ischemia-reperfusion injury. In addition, chromatin immunoprecipitation analysis identified NF-*κ*B as a principal inducer of Notch3 in ischemia reperfusion. NF-*κ*B is pivotal for the macrophage activation [49, 50]. This group also found that renal macrophages derived from Notch3-knockout mice could not activate proinflammatory cytokines. These data indicate that targeting Notch3 may be a new therapeutic strategy for AKI by mediating proinflammatory signaling.

### 7.2. Polycystic Kidney Disease (PKD)

In patients with PKD, the kidneys contain multiple fluid-filled cysts, and other organs may also be affected. Autosomal dominant PKD (ADPKD) is the most common form of PKD and is found primarily in adults [51, 52]. Autosomal recessive PKD (ARPKD) is rarer and usually much more severe than ADPKD [53–56]. ARPKD is found mostly in children, and patients often die perinatally or in infancy. Both ADPKD and ARPKD are associated with substantial morbidity and mortality.

Data from ARPKD and ADPKD mouse models and ADPKD patients indicate that Notch3 is consistently upregulated in cyst-lining epithelial cells [57]. The Notch3 expression was found to correlate with rapidly growing cysts, and Notch3 was found to be colocalized with PCNA (a proliferation marker). More importantly, Notch signaling blockade significantly decreased the forskolin-induced cyst formation and growth in human ADPKD cells. These data suggest that Notch3 is aberrantly activated and facilitates epithelial cell proliferation in PKD. Moreover, Notch3 inhibitors might be a therapeutic option for PKD.

## 8. Notch3 and Renal Fibrosis

Renal fibrosis is the final process in chronic kidney disease (CKD), and effective therapies to prevent or reverse renal fibrosis are lacking. Tubular cell injury and fibroblast recruitment and activation are key promoters of renal tubulointerstitial inflammation and fibrosis [58]. Accumulating evidence supports a role of Notch3 in renal fibrosis [6, 9, 29]. Djudjaj et al. [29] found increased Notch3 expression in tubular cells, particularly in dilated tubules and some tubulointerstitial cells, in a murine model of UUO, which is a widely used animal model for tubulointerstitial injury and damage and renal fibrosis. In addition, kidneys from Notch3-knockout mice seemed to exhibit protection against tubular damage and against tubular and interstitial proliferation, inflammation, and fibrosis induced by the UUO modeling. Moreover, Notch3-knockout mice exhibited less severe effects than wild-type mice after 14 days of the UUO modeling.

Huang et al. [9] reported that Notch3 is a potential contributor to renal interstitial fibrosis. This group proved their findings in clinical samples, animals, and cells. In humans, the increased expression of Notch3 was observed in renal tubular epithelial cells from patients with obstructive nephropathy compared to those of healthy individuals. In the rat model of UUO, which mimics human disease, Notch3 upregulation closely followed the processes of renal injury, renal fibrosis, TGF-*β* expression, and *α*-SMA expression. In human proximal tubule epithelial cells (HK-2 cells), TGF-*β* was found to promote ERK activation, which contributes to the Notch3 expression.

A study of human kidney biopsies from patients with obstructive nephropathy showed that the Notch3 expression was significantly increased in renal tubular epithelial cells [9]. Additionally, Notch3 may interact with other renal fibrosis signaling molecules. The high expression of TGF-*β*-Notch3 has been shown to be an important factor in promoting renal fibrosis [59], and inhibition of the Notch3 expression might be an effective therapeutic approach to alleviate renal interstitial fibrosis.

## 9. Conclusion and Prospects

The abnormal activation of Notch3 promotes the proliferation of a variety of cells, such as intrinsic glomerular cells, tubular epithelial cells, and interstitial fibroblasts, which mediate the occurrence and development of renal diseases (Table 1). Although inhibition of the Notch3 signaling pathway can alleviate renal damage, because of the significant inhibition of precursor cell proliferation during the disease repair process, Notch3 signaling pathway inhibition can instead lead to obstruction of renal repair and deterioration of renal function [34]. Due to the functional overlap and crosstalk between different subtypes of Notch receptors, researchers primarily use nonspecific *γ*-secretase inhibitors in studies, which leads to limitations and uncertainties in the final data. Recently, researchers have begun to use Notch3 antisense oligodeoxynucleotides (ODNs) to target the Notch3 activation, and specific antibodies against Notch signaling have been developed [60]. These tools help scholars to obtain not only more accurate information on the Notch3 signaling pathway but also more specific therapeutic measures to treat diseases related to Notch3 gene mutations. Future research should focus on canonical and noncanonical signaling pathways of Notch3 and the mutual regulation among Notch receptors and their downstream target genes. This knowledge would be helpful in treating Notch3 overactivation-related diseases.

However, the identity of the cell types in which Notch3 is activated in different renal diseases is an unsolved issue that might shed light on the effects of Notch3 signaling. In the animal model of UUO, Notch3 signaling is activated in tubular and interstitial cells. In contrast, in mouse models and patients with ADPKD, Notch3 is upregulated in cyst-lining epithelial cells. In lupus nephritis [41], rapidly progressive renal disease [20], and focal segmental glomerulonephropathy [34], Notch3 is expressed in glomerular podocytes. In summary, the identification of Notch3 target cells in the context of different types of renal injury is of vital importance.

## Figures and Tables

**Table 1:** Notch3 and renal diseases.

Notch3 source	Diseases
VSMCs	CADASIL
Podocytes	FSGS, lupus nephritis, RPGN
PECs	FSGS, lupus nephritis, RPGN
Mesangial cells	IgA nephropathy, membranoproliferative glomerulonephritis, or metabolic diseases (such as diabetes)
Renal epithelial cells	AKI, obstructive nephropathy
Cyst-lining epithelial cells	PKD

VSMCs: vascular smooth muscle cells; CADASIL: cerebral autosomal dominant arteriopathy with subcortical infarcts and leukoencephalopathy; FSGS: focal segmental glomerulosclerosis; RPGN: rapidly progressive glomerulonephritis; AKI: acute kidney injury; PKD: polycystic kidney disease.

## Data Availability

The data used to support the findings of this study are available from the corresponding author upon request.
